# Early-onset neonatal sepsis and antibiotic use in Indonesia: a descriptive, cross-sectional study

**DOI:** 10.1186/s12889-022-13343-1

**Published:** 2022-05-17

**Authors:** Khansa Salsabila, Nadira Mohammad Ali Toha, Lily Rundjan, Porjai Pattanittum, Prapassara Sirikarn, Rinawati Rohsiswatmo, Setya Wandita, Mohammad Hakimi, Pagakrong Lumbiganon, Sally Green, Tari Turner

**Affiliations:** 1 Faculty of Medicine, Universitas Indonesia, Jakarta, Indonesia; 2 School of Public Health and Preventive Medicine, Monash University, 3rd Floor, 553 St Kilda Road, Melbourne, VIC 3004 Australia; 3 Department of Child Health, Faculty of Medicine, Universitas Indonesia - Cipto Mangunkusumo Hospital, Jakarta, Indonesia; 4 Department of Epidemiology and Biostatistics, Faculty of Public Health, Khon Kaen, Thailand; 5 Department of Child Health, Dr Sardjito Hospital, Yogyakarta, Indonesia; 6 Department of Obstetrics and Gynaecology, Gadjah Mada University, Yogyakarta, Indonesia; 7 Department of Pediatrics, Khon Kaen University, Khon Kaen, Thailand

**Keywords:** Early-onset sepsis, Neonate, Antibiotic use, Indonesia

## Abstract

**Background:**

Early diagnosis and prompt antibiotic treatment are crucial to reducing morbidity and mortality of early-onset sepsis (EOS) in neonates. However, this strategy remains challenging due to non-specific clinical findings and limited facilities. Inappropriate antibiotics use is associated with ineffective therapy and adverse outcomes. This study aims to determine the characteristics of EOS and use of antibiotics in the neonatal-intensive care units (NICUs) in Indonesia, informing efforts to drive improvements in the prevention, diagnosis, and treatment of EOS.

**Methods:**

A descriptive study was conducted based on pre-intervention data of the South East Asia-Using Research for Change in Hospital-acquired Infection in Neonates project. Our study population consisted of neonates admitted within 72 h of life to the three participating NICUs. Neonates who presented with three or more clinical signs or laboratory results consistent with sepsis and who received antibiotics for 5 consecutive days were considered to have EOS. Culture-proven EOS was defined as positive blood or cerebrospinal fluid culture. Type and duration of antibiotics used were also documented.

**Results:**

Of 2,509 neonates, 242 cases were suspected of having EOS (9.6%) with culture-proven sepsis in 83 cases (5.0% of neonatal admissions in hospitals with culture facilities). The causative organisms were mostly gram-negative bacteria (85/94; 90.4%). Ampicillin / amoxicillin and amikacin were the most frequently prescribed antibiotics in hospitals with culture facilities, while a third-generation cephalosporin was mostly administered in hospital without culture facilities. The median durations of antibiotic therapy were 19 and 9 days in culture-proven and culture-negative EOS groups, respectively.

**Conclusions:**

The overall incidence of EOS and culture-proven EOS was high in Indonesia, with diverse and prolonged use of antibiotics. Prospective antibiotic surveillance and stewardship interventions are required.

## Background

Neonatal sepsis is a bloodstream infection that occurs in the first 28 days of life, and is classified into early-onset (EOS) and late-onset (LOS) sepsis. Early-onset sepsis appears within the first 48–72 h of life, while LOS occurs beyond 72 h after birth [1, 2]. Pathogens are transmitted vertically prior to or during delivery in EOS, whereas LOS is primarily associated with horizontal transmission of pathogen**s **from hospital environment or invasive procedures [2–4]. The incidence of EOS and spectrum of causative organisms varies between countries and neonatal units. Low- to middle-income countries (LMICs) have a higher incidence of clinical EOS ranging from 20.7 to 39.3 per 1000 live births [5–8]. Up until now, Indonesia has not had a national registry for the incidence of EOS. The most common causative agents of EOS in high-income countries (HICs) are Group B *Streptococcus* (GBS) and *E.coli *[9, 10], while in LMICs, EOS might be dominated by gram-negative bacteria [11, 12], with these organisms being associated with more significant morbidity and higher mortality. Early diagnosis and treatment are crucial to reduce the burden of serious infection. A higher mortality rate is often reported in EOS as compared to LOS, highlighting the importance of EOS in our study [13–15].

Antibiotic therapy is crucial in management of neonatal sepsis and should be administered empirically to infants when there is clinical suspicion of infection. Due to the non-specific clinical findings and limited diagnostic facilities, this treatment approach remains challenging in LMICs, with possible risk of either under- or overtreatment. Inappropriate use of antibiotics may lead to ineffective treatment, with risk of exposure to medication side effects and the development of antimicrobial resistance (AMR) [16, 17]. Several studies have reported an increasing trend of AMR in both HICs and LMICs [5, 10, 18–21]. More recently, concerns have also been raised about the emergence of multi-drug resistant pathogens in the neonatal units of LMICs [20–24]. Both surveillance of pathogen and antibiotic sensitivities, which differ from one unit to another, play an important role in the establishment of appropriate empiric treatment. However, due to limited resources, this information is still not available in most neonatal units in Indonesia.

The aim of this study is to describe the characteristics of EOS and use of antibiotics in the neonatal-intensive care units (NICUs) of three Indonesian hospitals participating in the South East Asia-Using Research for Change in Hospital Acquired Infection in Neonates (SEA-URCHIN) project.

## Methods

The SEA-URCHIN project was an interrupted time series study which focused on decreasing neonatal mortality and infection in four Southeast Asian countries: Indonesia, Thailand, Malaysia, and Philippines. The project had three main phases (pre-intervention, intervention, and post-intervention period), each operating for one year. Data was collected during the pre- and post-intervention period. In this study, a secondary analysis was conducted on data extracted during pre-intervention period (June 2012 – May 2013) from three participating hospitals in Indonesia. The results of the SEA-URCHIN study will be reported separately.

The SEA-URCHIN project was undertaken in Level 2 and 3 neonatal units in three hospitals in Indonesia, consisting of two University Hospitals (National and Provincial) and one District Hospital. SEA-URCHIN aimed to recruit 100 neonates from each hospital every month for 12 months. Relevant data regarding clinical practices and outcomes of these neonates were obtained from medical records and entered into standardized case record forms by research assistants (medical doctors) in the National Hospital and nurses in the Provincial and the District Hospital. Additional antibiotic record forms, completed by research assistants or nurses, were provided for neonates who received antibiotic therapy for at least three days. This data included predefined clinical and laboratory data suggestive of sepsis, reasons for antibiotic administration, and a record of clinical outcomes, such as mortality, and assessment of the cause of death by neonatologists, if relevant.

The study population consisted of neonates admitted to the NICUs within the first three days of life. These neonates were identified as having EOS if they had at least three clinical signs and/or laboratory results suggesting EOS and were commenced on antibiotics within the first three days of life which were then continued for at least five consecutive days. Those who received antibiotics within the first three days of life but did not meet the set criteria for EOS were considered as non-EOS. All infants who were admitted after the first three days of life, or who had missing date for birth or admission, or data errors in date of antibiotic administration or admission, were excluded from the study.

The predefined clinical signs and laboratory results considered suggestive of sepsis included: (1) increased ventilator support or oxygen requirement, (2) increase in apnoea or bradycardia episodes or tachycardia, (3) prolonged capillary refill time or hypotension, (4) lethargy, (5) temperature instability, (6) abdominal distension or feeding intolerance/ileus, (7) glucose intolerance, and (8) base deficit greater than 10 mmol/L [1].

EOS was further categorized into either culture-proven or culture-negative EOS. Culture-proven EOS was defined as when a pathogen grew from either blood or cerebrospinal fluid (CSF) cultures [25]. However, if *Bacillus* species, diphtheroids or coagulase-negative *Staphylococcus*(CoNS) were observed in a single blood culture from neonates who did not receive appropriate antibiotic treatment but still had good outcomes, these were deemed to be contaminants. The episode was then defined as culture-negative EOS or clinical sepsis [2, 3].

Data about antibiotic use in the admitted neonates are presented as initial and overall use. The duration of antibiotic use was measured in days, starting from the first day of treatment, extending to and including the day of discontinuation of all antibiotics. Antibiotics administered for prophylaxis were not included in this study.

### Data analysis

Estimated EOS rates in the NICUs were obtained by dividing the number of neonates with EOS by the total number of neonates admitted to the participating NICUs within 3 days of life. The 95% confidence interval (CI) was calculated using Poisson distribution. The incidence of EOS was also estimated by dividing the number of inborn neonates with EOS by the total number of live births in the participating hospitals. Statistical analysis was performed using STATA version 15.0.

Ethics approval for the SEA-URCHIN project was obtained from the Monash University Human Research Ethics Committee (MUHREC) (CF11/2221–2011001241) following ethics approval and the letters of permission from each of the 11 hospitals in South East Asia participating in the project. This secondary analysis was approved by the research ethical clearance committee of all participating hospitals in Indonesia and the MUHREC (Project ID 19090).

## Results

**Fig. 1:**
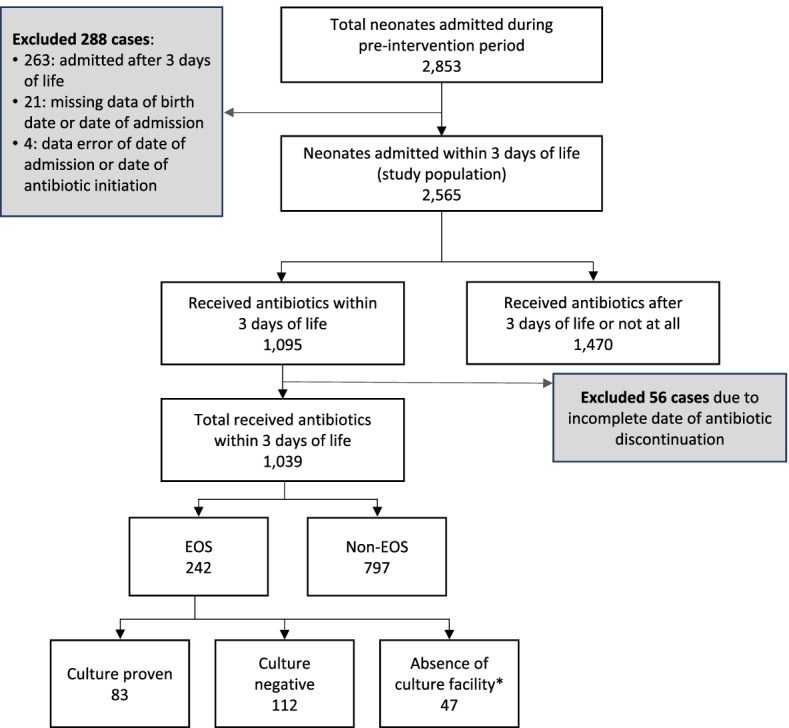
Study Flow. *No culture facility only in the District Hospital

During the pre-intervention period, there were 2,853 admissions to the three participating NICUs, with 2,565 neonates admitted in the first 3 days of life. These neonates formed our study population (Fig. 1). From this study population, a total of 1,039 (41.4%) neonates who received antibiotics within the first 3 days of life were analysed. There were 242 (23.3%) neonates who met criteria of EOS and of these, 195 (80.6%) had a blood culture collected. Positive blood cultures were noted in 83 (42.6%) of these infants.

Baseline maternal and neonatal characteristics in each hospital are shown in Table 1 and Table 2. A majority of the neonates were inborn (95.4%) with a mean gestational age of 36.8 ± 3.5 weeks and a mean birth weight of 2,543.4 ± 765.6 g. Of 2,498 mothers, there were 318 (12.7%) cases of premature rupture of membranes (PROM) and 165 (6.6%) cases of preterm premature rupture of membranes (PPROM). Approximately half of mothers received antibiotics within the 48 h before delivery, with PROM > 12 h and maternal fever in labour as being the most common reasons after excluding prophylactic use of antibiotics in caesarean section.

**Table 1:** Baseline maternal characteristics of admitted neonates in three Indonesian NICUs within three days of life

Characteristics^†^	National Hospital (*n* = 802)	Provincial Hospital (*n* = 879)	District Hospital (*n* = 884)	Total (*n* = 2,565)
**Maternal data**				
Number of mothers	799	849	855	2,503
Maternal age, year, mean (SD) (*n* = 2,414)	29 (6.6)	30 (6.7)	29.7 (6.5)	29.6 (6.6)
**Multiple pregnancy, n (%) (*n* = 2,491)**				
Singleton	707 (89.2)	797 (94.0)	820 (96.5)	2,324 (93.3)
Twins	78 (9.8)	51 (6.0)	30 (3.5)	159 (6.4)
Triplets	8 (1.0)	0 (0.0)	0 (0.0)	8 (0.3)
**PROM, n (%) (*n* = 318 in 2,498)**				
< 12 h	44 (5.5)	2 (0.2)	14 (1.6)	60 (2.4)
12 – 23 h	41 (5.2)	6 (0.7)	96 (11.2)	143 (5.7)
> 24 h	37 (4.7)	3 (0.4)	75 (8.8)	115 (4.6)
**PPROM (GA < 37 wk), n (%) (*n* = 165 in 2,498)**				
< 12 h	38 (4.8)	1 (0.1)	0 (0.0)	39 (1.6)
12 – 23 h	26 (3.3)	0 (0.0)	6 (0.7)	32 (1.3)
> 24 h	73 (9.2)	5 (0.6)	16 (1.9)	94 (3.8)
**Mode of delivery of first infant, n (%) (n = 2,490)**				
Normal labour	257 (32.6)	407 (47.9)	446 (52.3)	1,110 (44.6)
Vaginal breech	5 (0.6)	7 (0.8)	12 (1.4)	24 (1.0)
Emergency C section	384 (48.7)	259 (30.5)	126 (14.8)	769 (30.9)
Elective C section	102 (12.9)	140 (16.5)	202 (23.7)	444 (17.8)
Vacuum extraction	29 (3.7)	36 (4.2)	67 (7.9)	132 (5.3)
Forceps	11 (1.4)	0 (0.0)	0 (0.0)	11 (0.4)
**Antibiotic < 48 h before delivery (*n* = 1,055 in 2,096)**				
**Most common reasons for antibiotics administration, n (%)**				
Caesarean section	343	359	319	1,021
PROM > 12 h	118	31	107	256
Fever in labour	17	2	2	21

^†^Percentage for each characteristic was calculated from study population (mothers or neonates as specified) after excluding cases with missing data or where the variable was coded as ‘unknown’.

**Table 2:** Baseline characteristics of admitted neonates in three Indonesian NICUs within three days of life

Characteristics^†^	National Hospital (*n* = 802)	Provincial Hospital (*n* = 879)	District Hospital (*n* = 884)	Total (*n* = 2,565)
**Neonatal data**				
**GA at delivery, weeks, n (%) (*n* = 2,433)**				
> 41	3 (0.4)	7 (0.8)	15 (1.8)	25 (1.0)
> 37—< 41	346 (44.4)	495 (59.7)	704 (85.4)	1,546 (63.5)
> 32—< 37	329 (42.2)	237 (28.6)	81 (9.8)	647 (26.6)
< 32	101 (13.0)	90 (10.9)	24 (2.9)	215 (8.8)
Mean (SD)	35.4 (3.6)	36.4 (3.6)	38.5 (2.5)	36.8 (3.5)
**Birth weight, grams, n (%) (*n* = 2,526)**				
> 2,500	325 (42.3)	456 (51.9)	652 (74.3)	1.433 (56.7)
1,500 – 2,499	302 (39.3)	319 (36.3)	202 (23.0)	823 (32.6)
1,000 – 1,499	112 (14.6)	76 (8.7)	17 (1.9)	205 (8.1)
< 1,000	30 (3.9)	28 (3.2)	7 (0.8)	65 (2.6)
Mean (SD)	2,288.2 (817.5)	2,505.2 (789.4)	2,805.2 (592.7)	2,543.4 (765.6)
**Gender, n (%) (*n* = 2,553)**				
Male	433 (54.5)	457 (52.2)	461 (52.1)	1,351 (52.9)
Female	361 (45.5)	418 (47.8)	423 (47.9)	1,202 (47.1)
**Admission type, n (%) (n = 2,491)**				
Inborn	743 (95.4)	755 (88.7)	844 (98)	2,342 (94.0)
Outborn	36 (4.6)	96 (11.3)	17 (2.0)	149 (6.0)
**Apgar score at 5 min, n (%) (*n* = 2,450)**				
> 7	633 (82.1)	743 (90.1)	796 (93.2)	2,172 (88.7)
< 7	138 (17.9)	82 (9.9)	58 (6.8)	278 (11.3)
**Resuscitation (*n* = 1,196 in 2,488)**				
**Invasive procedures, n (%)**				
Oro/nasogastric tube	648 (80.8)	450 (51.2)	212 (24.0)	1,310 (51.1)
Exchange/blood transfusion	69 (8.6)	151 (17.2)	6 (0.7)	226 (9.0)
Urine catheter	18 (2.2)	16 (1.8)	0 (0.0)	34 (1.3)
Endotracheal tube	12 (1.5)	0 (0.0)	0 (0.0)	12 (0.5)

^†^Percentage for each characteristic was calculated from study population (mothers or neonates as specified) after excluding cases with missing data or where the variable was coded as ‘unknown’.

During our study period, the incidence rate of EOS in the combined data from the three hospitals was 26.6 per 1000 live births (202 inborn with EOS / 7,590 live births; 95% CI: 23.2–30.5) or 9.6% (242 EOS / 2509 admitted neonates; 95% CI: 8.5—10.9) of admitted neonates. The incidence of culture-proven EOS was 11.5 per 1000 live births (95% CI: 9.0–14.7) or 5% (95% CI: 4.1–6.2) of admitted neonates.

Rates of EOS based on each important characteristic in the 2,509 neonates admitted to the NICU within 3 days of life are presented in Table 3. These data demonstrated that a higher rate of EOS was seen in neonates who were outborn (19.2%; 95% CI: 13.7–26.2), with 47.5% (19/40; 95% CI: 34.3–65.8) being culture-proven. Early-onset sepsis was also more frequent among infants who were very preterm (29.2%; 95% CI: 22.2–37.7), extremely low birth weight (ELBW) (37.9%; 95% CI: 23.8–57.4), born to a mother with PROM ≥ 24 h (4.4%; 95% CI: 1.4–10.3) or PPROM < 12 h (25.6%; 95% CI: 12.3–47.2), and who had a low APGAR score (< 7) at 5 min (22.7%; 95% CI: 17.3–29.3).

**Table 3:** Frequencies of EOS based on the characteristics of 2,509 neonates admitted within 3 days of life

Characteristics^a^	Total admitted neonates	EOS				% (95% CI)
		**Culture proven (*n* = 83)**	**Culture negative (*n* = 112)**	**Absence of culture facility (*n* = 47)**	**Total (*n* = 242)**	
**Admission type (*n* = 2,506)**						
Inborn	2,298	64	95	43	202	8.8 (7.6–10.1)
Outborn	208	19	17	4	40	19.2 (13.7–26.2)
**Gender (*n* = 2,497)**						
Female	1,176	35	49	16	100	8.5 (6.9–10.3)
Male	1,321	48	63	31	142	10.7 (9.1–12.7)
**GA at birth, weeks (*n* = 2,380)**						
> 41	24	0	0	1	1	4.2 (0.1–23.2)
> 37—< 41	1,515	19	28	26	73	4.8 (3.8–6.1)
> 32—< 37	639	38	47	10	95	14.9 (12.0–18.2)
< 32	202	20	32	7	59	29.2 (22.2–37.7)
**Birth weight, grams (*n* = 2,470)**						
> 2,500	1,399	17	26	19	62	4.4 (3.4–5.7)
1,500 – 2,499	813	38	41	19	98	12.1 (9.8–14.7)
1,000 – 1,499	200	22	31	5	58	29.0 (22.0–37.5)
< 1,000	58	5	14	3	22	37.9 (23.8–57.4)
**Birth asphyxia APGAR at 5 min (*n* = 2,398)**						
> 7	2,134	64	74	31	169	7.9 (6.8–9.2)
< 7	264	16	31	13	60	22.7 (17.3–29.3)
**Maternal PROM, hours (*n* = 315)**						
< 12	60	1	0	1	2	3.3 (0.4–12.0)
12—23	142	0	1	3	4	2.8 (0.8–7.2)
> 24	113	0	3	2	5	4.4 (1.4–10.3)
**Maternal PPROM, hours (*n* = 165)**						
< 12	39	1	9	0	10	25.6 (12.3–47.2)
12—23	32	1	2	2	5	15.6 (5.1–36.5)
> 24	94	0	11	2	13	13.8 (7.4–23.6)

Abbreviation: *APGAR* appearance, pulse, grimace, activity, and respiration, *GA* gestational age, *PPROM* preterm premature rupture of membranes, *PROM* premature rupture of membrane

^a^Percentage for each characteristic was calculated from study population (mothers or neonates as specified) after excluding cases with missing data or where the variable was coded as ‘unknown’

Among neonates with positive cultures, the majority organisms were Gram-negative bacteria (85/94; 90.4%), including *Burkholderia cepacia* (50/94; 52.1%), *Klebsiella pneumoniae* (9/94; 9.4%) and *Pseudomonas aeruginosa* (6/94; 6.3%). This group of pathogens were only identified in the Provincial Hospital. In comparison, there were 7 positive blood cultures in the National Hospital, with *Acinetobacter Sp.* as the most frequent pathogen (2/7).

The clinical and laboratory characteristics, outcomes and causes of death in the EOS and non-EOS groups are shown in Table 4. Increased oxygen requirement (81.4%), temperature instability (78.1%), and lethargy (64.0%) were the three most common clinical manifestations noted in neonates with EOS. A similar pattern of clinical manifestations was also seen in the non-EOS group. Neonatal mortality in the EOS group within 28 days of life was 21.5%, which was higher than in the non-EOS group (10.2%). The 14-day mortality was higher in EOS group (17.4%) compared to the non-EOS group (9.8%), with the most common causes in the EOS group cardiorespiratory disorder (9.5%), infection (8.3%), and extreme prematurity (5.8%).

**Table 4:** Clinical, laboratory variables and outcomes in each group of neonates admitted within 3 days of life

**Clinical and laboratory variables**	**Received antibiotic within 3d of life (*n*** = **1,039)**				
	**EOS**				**Non-EOS** **(*n*** = **797)**
	**Culture proven** **(*n*** = **83)**	**Culture negative** **(*n*** = **112)**	**Absence of culture facility** **(*n*** = **47)**	**Total** **(*n*** = **242)**	
Temperature instability	68 (81.9%)	81 (72.3%)	40 (85.1%)	189 (78.1%)	219 (27.5%)
Increased oxygen requirement or ventilatory support	65 (78.3%)	88 (78.6%)	44 (93.6%)	197 (81.4%)	355 (44.5%)
Glucose intolerance	64 (77.1%)	56 (50.0%)	11 (23.4%)	131 (54.1%)	121 (15.2%)
Lethargy	62 (74.7%)	63 (56.3%)	30 (63.8%)	155 (64.0%)	133 (16.7%)
Ileus/feeding intolerance or abdominal distension	52 (62.7%)	48 (42.9%)	21 (44.7%)	121 (50.0%)	81 (10.2%)
Increase in apnoeic or bradycardic episodes or tachycardia	24 (28.9%)	38 (33.9%)	13 (27.7%)	75 (31.0%)	56 (7.0%)
Hypotension or prolonged capillary refill	5 (6.0%)	15 (13.4%)	12 (25.5%)	32 (13.2%)	31 (3.9%)
Base deficit > 10 mmol/L	5 (6.0%)	34 (30.4%)	0 (0.0%)	39 (16.1%)	62 (7.8%)
Outcomes					
14-d mortality	12 (14.5%)	27 (24.1%)	3 (6.4%)	42 (17.4%)	78 (9.8%)
28-d mortality	21 (25.3%)	28 (25.0%)	3 (6.4%)	52 (21.5%)	81 (10.2%)

**Table ****5** presents the type of antibiotics given as initial and overall therapy in each group of neonates.

**Table 5:** Antibiotic use in neonates (initial and overall) within 3 days of life

**Antibiotics**	**EOS**			**Non-EOS** **(*n*** = **797)**
	**Culture proven** **(*n*** = **83)**	**Culture negative** **(*n*** = **112)**	**Absence of culture facility** **(*n*** = **47)**	
Initial antibiotics				
Ampicillin / Amoxicillin	76 (91.6%)	47 (42.0%)	3 (6.4%)	156 (19.6%)
Gentamicin	17 (20.5%)	55 (49.1%)	10 (21.3%)	457 (57.3%)
Amikacin	78 (94.0%)	73 (65.2%)	2 (4.3%)	229 (28.7%)
Third-generation cephalosporin	27 (32.5%)	26 (23.2%)	41 (87.2%)	181 (22.7%)
Overall antibiotics				
Ampicillin / Amoxicillin	76 (91.6%)	48 (42.9%)	3 (6.4%)	157 (19.7%)
Penicillin	0 (0.0%)	0 (0.0%)	0 (0.0%)	1 (0.1%)
Gentamicin	19 (22.9%)	55 (49.1%)	10 (21.3%)	458 (57.5%)
Netilmicin	39 (47.0%)	8 (7.1%)	0 (0.0%)	12 (1.5%)
Amikacin	82 (98.8%)	74 (66.1%)	2 (4.3%)	229 (28.7%)
Ampicillin-Sulbactam	1 (1.2%)	0 (0.0%)	0 (0.0%)	0 (0.0%)
Amoxicillin-Clavulanic acid	3 (3.6%)	45 (40.2%)	0 (0.0%)	398 (49.9%)
Piperacillin-tazobactam	3 (3.6%)	28 (25%)	0 (0.0%)	93 (11.4%)
Imipenem	4 (4.8%)	1 (0.9%)	0 (0.0%)	0 (0.0%)
Meropenem	20 (24.1%)	9 (8.0%)	0 (0.0%)	16 (2.0%)
Doripenem	0 (0.0%)	0 (0.0%)	0 (0.0%)	3 (0.4%)
Third-generation cephalosporin	68 (81.9%)	28 (25.0%)	41 (87.2%)	182 (22.8%)
Fourth-generation cephalosporin	0 (0.0%)	0 (0.0%)	0 (0.0%)	2 (0.3%)
Ciprofloxacin	0 (0.0%)	0 (0.0%)	0 (0.0%)	1 (0.1%)
Vancomycin	1 (1.2%)	0 (0.0%)	3 (6.4%)	3 (0.4%)
Metronidazole	11 (13.3%)	5 (4.5%)	0 (0.0%)	15 (1.9%)

The most common initial antibiotics prescribed among neonates with EOS in hospitals with culture facility were ampicillin / amoxicillin and amikacin (63%, 95% CI 56.7–70.2%; 77.4%; 95% CI: 71.8–83.5% respectively). A third generation cephalosporin (87.2%, 95% CI: 78.2–97.3%) was the most common initial antibiotic in the hospital with no culture facilities. In contrast, in the non-EOS group, the most frequent initial antibiotics chosen were gentamicin, amikacin and a third-generation cephalosporin (Table 5). The median duration of antibiotic therapy for infants with culture-proven sepsis was 19 days (IQR, 5 to 47). In the culture-negative and non-EOS group, the median durations were 9 (IQR, 5 to 37) and 6 (IQR, 1 to 56) days, respectively. Neonates with EOS in hospital without culture facilities received antibiotic treatment for 8 (IQR, 5 to 92) days.

## Discussion

In this secondary analysis from the pre-intervention period of the SEA-URCHIN project, the incidence of EOS across the three NICUs in Indonesia was 26.6 per 1000 live births or 9.6% of admitted neonates. The incidence of culture-proven EOS among inborn infants was 11.5 per 1000 live births or 5.0% of neonatal admissions in 2 hospitals with culture facility. The 14- and 28-day mortality rate of EOS were 17.4% and 21.5%, respectively. The most common organisms isolated were *Burkholderia cepacia* (52.1%), followed by *Klebsiella pneumoniae* (9.4%) and *Pseudomonas aeruginosa* (6.3%)*.* Ampicillin / amoxicillin and amikacin were the most commonly prescribed initial antibiotics in the hospitals with culture facilities, whereas a third-generation cephalosporin was commonly used in the hospital without culture facilities. The median duration of antibiotic therapy for culture-proven EOS was 19 days (range 5 to 47 days), whilst in the culture-negative and non-EOS groups, there were 9 days and 6 days, respectively.

The incidence rate of EOS in this study was higher compared to that reported from Thailand, another participating country in the SEA-URCHIN project, which was 8.8 per 1000 live births [26]. The incidence rate of culture-proven EOS in admitted neonates was much higher than Thailand (0.2%; 4 / 1,897) [5, 26], although this rate may have been underestimated due to unavailability of data from the District Hospital. Higher rates of EOS in our study might be attributed to the following factors. First, the volume of blood cultures taken in all participating hospitals was at least 1 ml as recommended, which gives excellent sensitivity in detecting infants with even very low density bacteraemia [27]. Other possible factors were no standardized policy for screening for infections in asymptomatic pregnant women and poor antenatal care which might result in insufficient time for maternal antibiotic coverage prior to or during labour. Also, the antibiotics choice in mothers with risk factors for infections may not have covered gram-negative bacteria in EOS.

The 28-day mortality rate of EOS in our study (21.5%) was substantially higher compared to that of Thailand (1.9%) [26]. However, the more recent report from United Nations Children’s Fund (UNICEF) in 2018 has shown a decrease rate of neonatal mortality due to infection in Indonesia (12%) [28]. Additionally, the 14-day-mortality rate in new-born infants with culture negative EOS was higher when compared to infants with culture proven EOS. This may be associated with the higher number of very low birth weight (VLBW) and preterm infants, and also infants with low APGAR scores in the culture negative EOS group, who have expected greater relative risk of death.

Neonates with EOS may acquire infection in utero or during the intrapartum period. Risk factors for EOS include both maternal and neonatal factors. Maternal factors, such as chorioamnionitis or ascending infection, may lead to in utero infection [29, 30]. During labour, maternal risk factors such as PROM, vaginal colonization and frequent vaginal examination may increase vertical transmission of microorganisms [31]. Other factors such as urinary tract infection and vaginal discharge have been reported as additional maternal risk factors in developing countries [32]. In our study, notable maternal risk factors for EOS were PROM and PPROM, which are consistent with other studies [4, 8, 33–36].

Neonatal risk factors associated with EOS include prematurity, low birth weight and 5-min APGAR score < 7. Hayun et al. [37] observed a 13.45 and 4.9 fold increase in risk for EOS among premature and VLBW infants, respectively. In our study, more than half of neonates with signs of EOS were preterm (63.6%) or VLBW (73.6%), and almost 25% of them had low APGAR score at 5 min. Studies [38–40] have demonstrated that neonates with a low APGAR have an increased risk of various interventional procedures and poor adaptation to extra-uterine life, increasing their susceptibility to infection. Alternatively, in utero infection may initially activate excessive inflammatory responses, disrupting placental blood flow and subsequently leading to neonatal asphyxia [41]. Because the correlation of asphyxia and infection can be reciprocal, these findings should be interpreted with caution.

Among the 242 neonates who fulfilled criteria of EOS, 83 (34.3%) were culture-proven, with the majority of positive cultures recorded in the Provincial Hospital (76/83; 91.6%). Most pathogens identified in this study were gram-negative bacteria, similar to findings in other LMICs [42–44]. The microbial patterns are diverse among neonatal units in Indonesia. In our study, the predominant microorganism from the University Hospital (*Acinetobacter sp*) was different from the Provincial Hospital (*Burkholderia cepacia*, *Klebsiella pneumoniae* and *Pseudomonas aeruginosa)*. In neonatal units in Medan (Indonesia) the most prevalent pathogens were *Klebsiella pneumonia* and *Enterobacter sp *[45, 46], and in Denpasar (Indonesia) was *Serratia marcescens *[47]. In our study, more than half of culture-proven EOS cases were caused by *Bulkholderia cepacia*, which is generally a rare cause of sepsis in neonates. This motile gram-negative bacillus survives in moist environments, including antiseptics, disinfectants and other medical solutions, which subsequently become a potential source of transmission. Direct transmission from person-to-person has also been reported [48]. A retrospective study in India [49] showed that majority of EOS cases caused by *Bulkholderia cepacia* were hospital-acquired, rather than maternal origin. The high rate of *Bulkholderia*infection in the Provincial Hospital may be due to an outbreak during the study period. In our study, the predominance of gram-negative bacteria in early-onset infections leads to the hypothesis that EOS in the hospital in LMICs may be hospital-acquired rather than maternally acquired. Lack of intrapartum and postnatal standard infection control practices in LMICs increase the risk of hospital-acquired infections [50]. In addition, gram-negative bacteria such as *Klebsiella spp* and *Acinetobacter spp *have been reported as the most frequent cause of outbreaks in developing countries as they survive in contaminated containers of medication, solutions such as antiseptics, or other equipment [50].

Kiatchoosakun et al. reported growth of GBS in the majority of their cultures in Thailand [26]. Similarly, GBS was identified as the most common pathogen in HICs such as the UK, Australia and New Zealand [9, 51], while *Staphylococcus aureus *most frequently seen in Norway and Denmark [52]. In our study, GBS was not detected in any of our cultures, and to date, there has been only 1 case reported in another Indonesian study [53]. Positive GBS colonization was reported in 31.3% and 16.4% of the pregnant women in Bali (2013) and Banda Aceh (2015) [54, 55]. However, because there is no GBS screening or antibiotic treatment policy for GBS-positive mothers in Indonesia, GBS might be underestimated in our study. Also, the GBS culture method has a false negative rate of up to 50%, dependent on the culture timing, swab location, culture method and culture media choice [56].

Clinical manifestations of EOS in neonates are non-specific and vary by gestational age and severity of illness [29]. The common clinical manifestations of EOS in our study were increased oxygen requirement or ventilator support, temperature instability and lethargy. Among these features, increased oxygen requirement or ventilator support occurred most frequently, which is similar to findings in other studies [26, 29, 57, 58]. Because there were no differences in clinical findings between the EOS and non-EOS groups, we recommend that the decision to start antibiotic treatment should not be based on the presence of clinical manifestations alone.

Treatment guidelines published by the World Health Organization (WHO), the National Institute for Health and Care Excellence (NICE), and the American Academy of Pediatrics (AAP) suggest use of a combination of the narrow-spectrum agents, penicillin and an aminoglycoside, as the first line therapy for EOS [59–61]. In contrast to these guidelines, wide variation in choice of empirical antibiotic regiments has been reported in several studies [62, 63]. Studies in HICs showed strong adherence to these guidelines [52, 64], whilst reports from Bangladesh, China and India demonstrated a high variety of antibiotics used for EOS [65]. In the present study, a majority of neonates with EOS in hospitals with culture facilities were initially prescribed ampicillin / amoxicillin and amikacin. In contrast, broad spectrum antibiotics such as a third generation cephalosporin was used as the first line of treatment in the hospital lacking culture facilities. Consistent with other LMICs, it is evident that a wide variety of broad spectrum antibiotics was prescribed as an empiric therapy in the 3 participating hospitals in Indonesia. Similarly, a study in Manado (Indonesia) reported use of a combination of ceftazidime and amikacin as the most common prescribed antibiotics in their NICU [66]. The reasons for this practice may be related to unclear guidelines for management of initial EOS in some hospitals, increased emergence of multidrug resistant pathogens, and unavailability of an antimicrobial stewardship team in the hospital during the study period.

In 2011, the Kaiser Permanente EOS calculator was developed based on maternal data such as intrapartum temperature, use of intrapartum antibiotics, duration of rupture of membrane, maternal GBS status, as well as neonatal factors such as gestational age and clinical exam findings. The calculator aims to limit the number of infants unnecessarily commenced on antibiotics for EOS, thereby minimizing the risk of antibiotic resistance [67]. In our study, more than 75% of our newborns who received antibiotics within the first 3 days of life were considered as non-EOS cases. This unnecessary antibiotic administration could have been reduced by using a screening method including the sepsis calculator. However, because maternal GBS status in Indonesia was unavailable during the study period, it would be difficult to apply the sepsis calculator in the Indonesian setting.

The AAP guidelines recommend that antibiotics should be given for at least 10 days in culture-proven sepsis and antibiotic use be re-evaluated by 48 h in neonates with negative culture or low probability of sepsis [61]. In our study, the median duration of antibiotic therapy was 19 and 9 days in culture-proven and culture-negative sepsis respectively, similar to that reported in Thailand [26]. The prolonged duration of antibiotic treatments in this study might be due to difficulty in differentiating between persisting symptoms due to non-response related to AMR or because of new onset of LOS. Because repeated blood culture was not routinely done except in the National Hospital, it is challenging to differentiate non-responsive EOS from the new onset of LOS. Most hospitals in Indonesia have limited resources to investigate and provide microbial and antimicrobial susceptibility patterns, hence the data about the impact of multi-drug resistance cases was also limited. Combined, these factors may contribute to the longer duration of antibiotic therapy reported in our study.

## Significance

Our findings provide data needed to drive initial improvements in all three areas of prevention, diagnosis, and treatment of EOS in Indonesia, including updated data from a large sample of infants in three NICUs in two regions of Indonesia, thereby forming the starting point for the development of an Indonesian AMR action plan.

Additional analysis could compare the post-intervention data from the SEA-URCHIN project with our pre-intervention data findings. The comparison between the two different periods could determine if the SEA-URCHIN interventions impacted on the three Indonesian hospitals, thereby improving our understanding of how infection contributes to neonatal mortality and morbidity amongst high-risk groups. This will lead to more effective efforts in prevention of sepsis, a reduction in mortality and the prevention of long-term morbidity for those who survive.

## Conclusions

The overall incidence of EOS and culture-proven EOS was high in our study, with a 28-day-neonatal mortality rate of 21.5%. In contrast to the guideline from WHO, NICE, and AAP, the initial antibiotics used in our study showed greater variation and longer duration than recommended.

Neonatal sepsis is preventable. Development of strategies for prevention should involve the healthcare providers as well as health policy makers to optimize prevention, early diagnosis and prompt treatment. Prospective antibiotic surveillance and stewardship interventions are required to reduce unnecessary antibiotic exposure in our NICU. To our knowledge, this study is the first prospective study describing the EOS incidence, its characteristics, and antibiotic use in Indonesia. Further follow-up studies are necessary for better understanding of EOS characteristics and antibiotic use in Indonesia.

## Data Availability

The datasets generated and analyzed during the current study are not publicly available due to the nature of the data, but are available from the corresponding author on reasonable request.

## Abbreviations

AAP: American Academy of Pediatrics
AMR: Antimicrobial Resistance
APGAR: Appearance, Pulse, Grimace, Activity, Respiration
CI: Confidence Interval
CoNS: Coagulase-Negative Staphylococci
CSF: Cerebrospinal Fluid
ELBW: Extremely Low Birth Weight
GBS: Group B Streptococcus
EOS: Early Onset Sepsis
HIC: High-Income Countries
LMIC: Low- to Middle-income Countries
LOS: Late Onset Sepsis
MUHREC: Monash University Human Research Ethics Committee
NICE: National Institute for Health and Care Excellence
NICU: Neonatal Intensive Care Unit
PPROM: Preterm Premature Rupture of Membranes
PROM: Premature Rupture of Membranes
SEA-URCHIN: South-East Asia—Using Research for Change in Hospital-acquired Infections in Neonates
UNICEF: United Nations Children’s Fund
VLBW: Very Low Birth Weight
WHO: World Health Organization
